# Preferences for a Mobile HIV Prevention App for Men Who Have Sex With Men

**DOI:** 10.2196/mhealth.3745

**Published:** 2014-10-29

**Authors:** Tamar Goldenberg, Sarah J McDougal, Patrick S Sullivan, Joanne D Stekler, Rob Stephenson

**Affiliations:** ^1^Rollins School of Public HealthDepartment of EpidemiologyEmory UniversityAtlanta, GAUnited States; ^2^Department of MedicineDivision of Allergy and Infectious DiseasesUniversity of WashingtonSeattle, WAUnited States; ^3^Rollins School of Public HealthHubert Department of Global HealthEmory UniversityAtlanta, GAUnited States

**Keywords:** MSM, HIV, mobile phone app, mHealth, HIV prevention

## Abstract

**Background:**

The Centers for Disease Control and Prevention recommends that sexually active men who have sex with men (MSM) in the United States test for human immunodeficiency virus (HIV) at least three times per year, but actual testing frequency is much less frequent. Though mHealth is a popular vehicle for delivering HIV interventions, there are currently no mobile phone apps that target MSM with the specific aim of building an HIV testing plan, and none that focuses on developing a comprehensive prevention plan and link MSM to additional HIV prevention and treatment resources. Previous research has suggested a need for more iterative feedback from the target population to ensure use of these interventions.

**Objective:**

The purpose of this study is to understand MSM’s preferences for functionality, format, and design of a mobile phone-based HIV prevention app and to examine MSM’s willingness to use an app for HIV prevention.

**Methods:**

We conducted focus group discussions with 38 gay and bisexual men, with two in-person groups in Atlanta, two in Seattle, and one online focus group discussion with gay and bisexual men in rural US regions. These discussions addressed MSM’s general preferences for apps, HIV testing barriers and facilitators for MSM, and ways that an HIV prevention app could address these barriers and facilitators to increase the frequency of HIV testing and prevention among MSM. During focus group discussions, participants were shown screenshots and provided feedback on potential app functions.

**Results:**

Participants provided preferences on functionality of the app, including the type and delivery of educational content, the value of interactive engagement, and the importance of social networking as an app component. Participants also discussed preferences on how the language should be framed for the delivery of information, identifying that an app needs to be simultaneously fun and professional. Privacy and altruistic motivation were considered to be important factors in men’s willingness to use a mobile HIV prevention app. Finally, men described the potential impact that a mobile HIV prevention app could have, identifying individual, interpersonal, and community-based benefits.

**Conclusions:**

In summary, participants described a comprehensive app that should incorporate innovative ideas to educate and engage men so that they would be motivated to use the app. In order for an app to be useful, it needs to feel safe and trustworthy, which is essential when considering the app’s language and privacy. Participants provided a range of preferences for using an HIV prevention app, including what they felt MSM need with regards to HIV prevention and what they want in order to engage with an app. Making an HIV prevention app enjoyable and usable for MSM is a difficult challenge. However, the usability of the app is vital because no matter how great the intervention, if MSM do not use the app, then it will not be useful.

## Introduction

Gay, bisexual, and other men who have sex with men (MSM) account for a disproportionate burden of incident human immunodeficiency virus (HIV) infections in the United States. Although MSM make up less than 2% of the population, in 2011, 62% of new HIV infections in the United States were among MSM [1]. In order to address this, the Centers for Disease Control and Prevention (CDC) recommends that MSM test for HIV infection at least three to four times per year [2]. Identification of new HIV infections is the first step in the cascade of HIV care [3]; however, less than 20% of MSM are testing at least three times per year [4]. HIV interventions that use multipronged approaches and incorporate biomedical, behavioral, and structural strategies to target HIV prevention among MSM are most effective [5]. In an age of advancing technology and increasing mobile phone use [6,7], Internet-based interventions and mHealth (the use of mobile phones for medical or public health supported interventions) have become a popular vehicle for a variety of health interventions [6,8,9] and may be a useful mechanism for bringing multifaceted HIV prevention strategies to scale [10,11].

Most existing mHealth HIV interventions have used mobile technology through the use of short message service (SMS) texting to provide HIV risk-reduction messages [12-15] and to improve adherence to highly active antiretroviral treatment [16-21]; many of these SMS-based interventions have been proven to be effective [19]. HIV interventions using mobile phone apps are also becoming increasingly popular. In their evaluation of the availability of HIV-prevention mobile apps, Muessig et al identified 55 unique mobile apps that address HIV prevention and care; however, these apps were not frequently downloaded and were not highly rated by their users [22].

In order to increase the uptake and use of apps used for HIV prevention, Muessig et al suggests that app developers collect input from the target audience through a process that identifies app preferences and evaluates the app [22]. In response to this, we conducted formative qualitative research with MSM to understand likely scenarios for app use, to identify preferences regarding functionality, format, and design, and to determine MSM’s willingness to use an HIV prevention mobile app. This would be the first app to guide MSM in building a comprehensive prevention plan and link them to HIV testing, HIV prevention services, and treatment resources.

## Methods

### Study Population and Recruitment

This study was approved by the Emory University Institutional Review Board. From August-December 2013, we recruited gay and bisexual men using flyers and Facebook advertisements. Flyers were posted in venues in Atlanta and Seattle where gay and bisexual men frequent (eg, restaurants, bars, coffee shops, gyms). Facebook advertisements targeted men living in Atlanta, Seattle, and rural regions who reported being interested in men in their profiles. In Atlanta, men recruited through Facebook have been reported to be comparable behaviorally to men recruited through other venues [23]. Rural locations were determined by postal codes using the US Census Bureau’s data and definition of rural (ie, population density <1000 people/square mile) [24]. The flyers and advertisements provided a link to an online screening survey through SurveyGizmo to determine study eligibility. Eligibility criteria included (1) age 18 years or older, (2) male, (3) self-identification as gay or bisexual, (4) current residence in Atlanta, GA, Seattle, WA, or in a rural US county, (5) never having tested positive for an HIV test, and (6) having ever owned a mobile phone. Eligible participants were contacted to participate in a focus group discussion (FGD).

### Study Procedures

We completed four in-person FGDs (two in Atlanta and two in Seattle) and one online FGD (OFGD) [25] with rural men. The OFGD used a chatroom-based format using Adobe Connect, a real-time Web-based meeting client. Adobe Connect allows for participants to view a variety of customizable windows, including a window for discussion, where they can communicate and type responses to questions as though in a chatroom. Other windows allowed the moderator to share screenshots and poll participants on app preferences. Participants were also able to contact the moderator privately if they had questions or comments that they did not want to express to the group.

**Figure 1:**
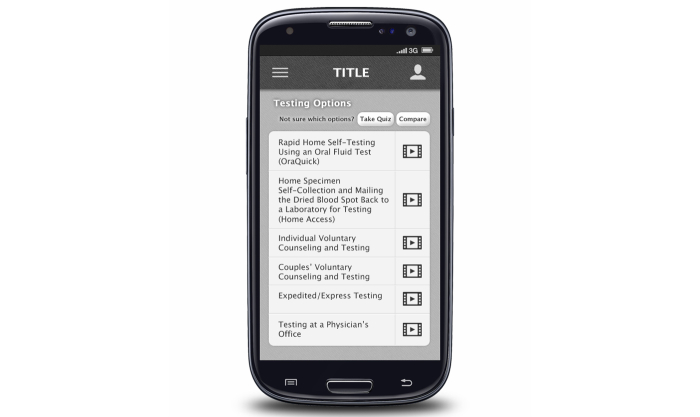
Screenshot for creating a testing plan.

**Figure 2:**
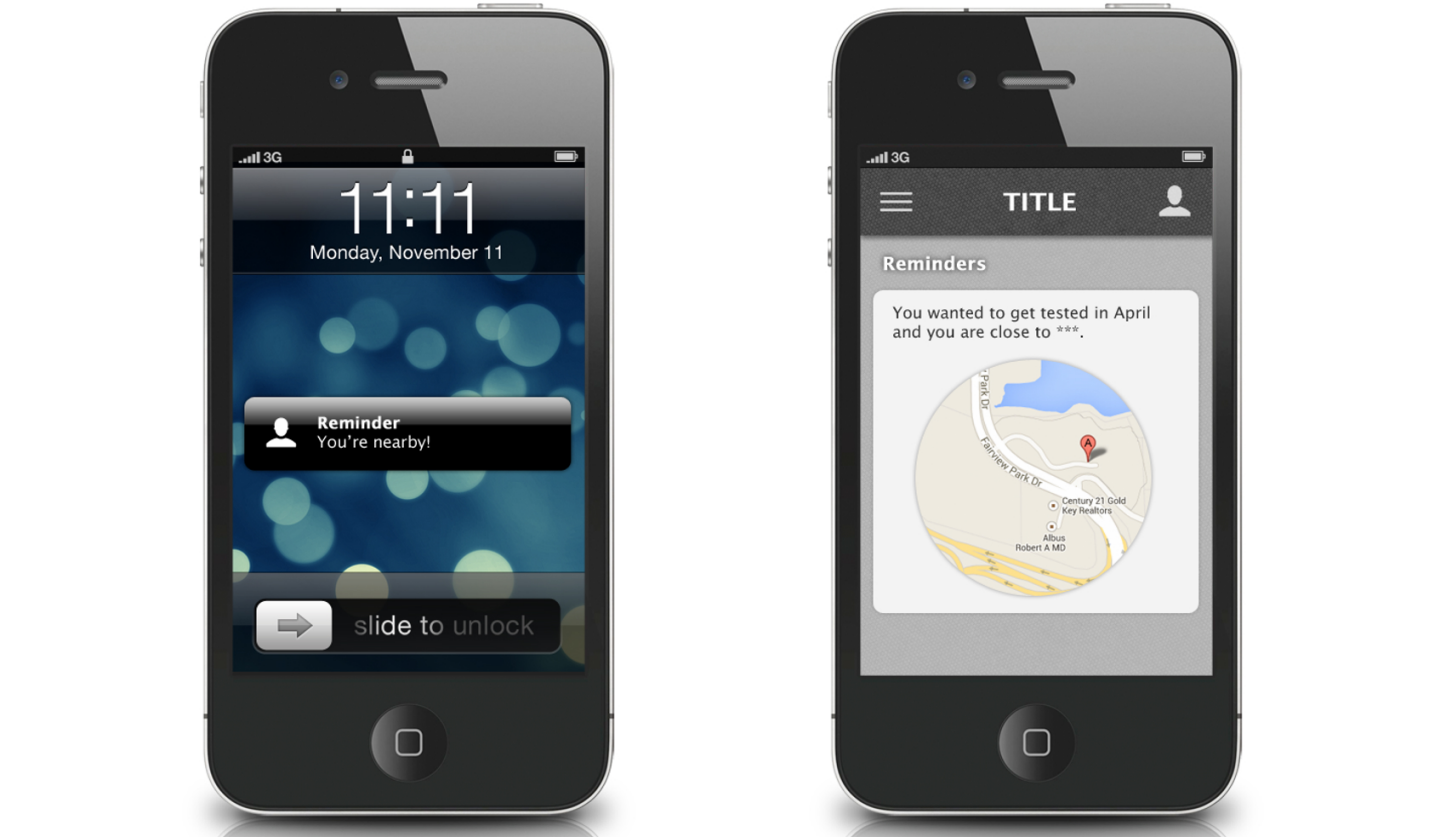
Screenshot for location-based reminders for HIV testing.

**Figure 3:**
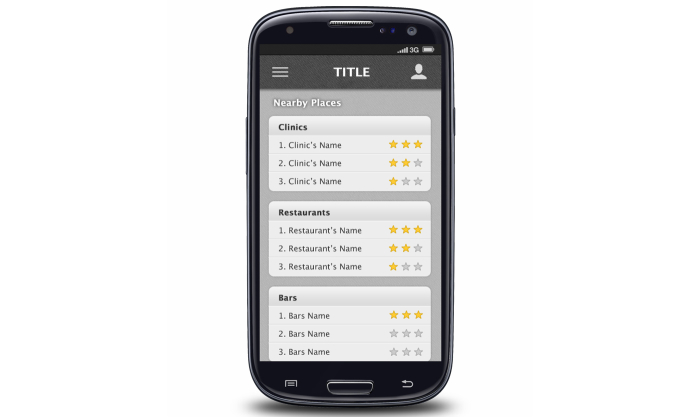
Screenshot for rating and reviewing HIV testing centers and other venues.

**Figure 4:**
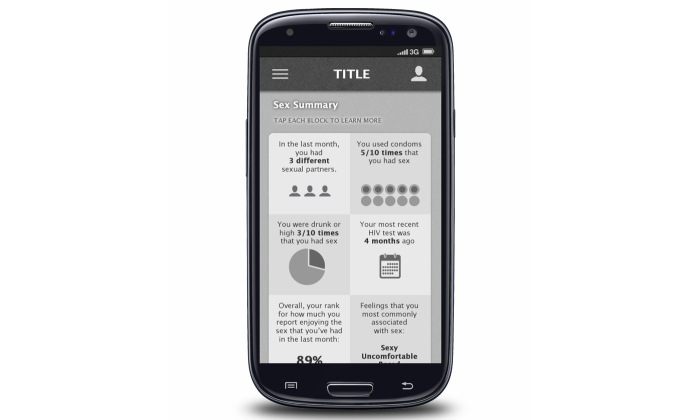
Screenshot for tracking of sexual behaviors.

**Figure 5:**
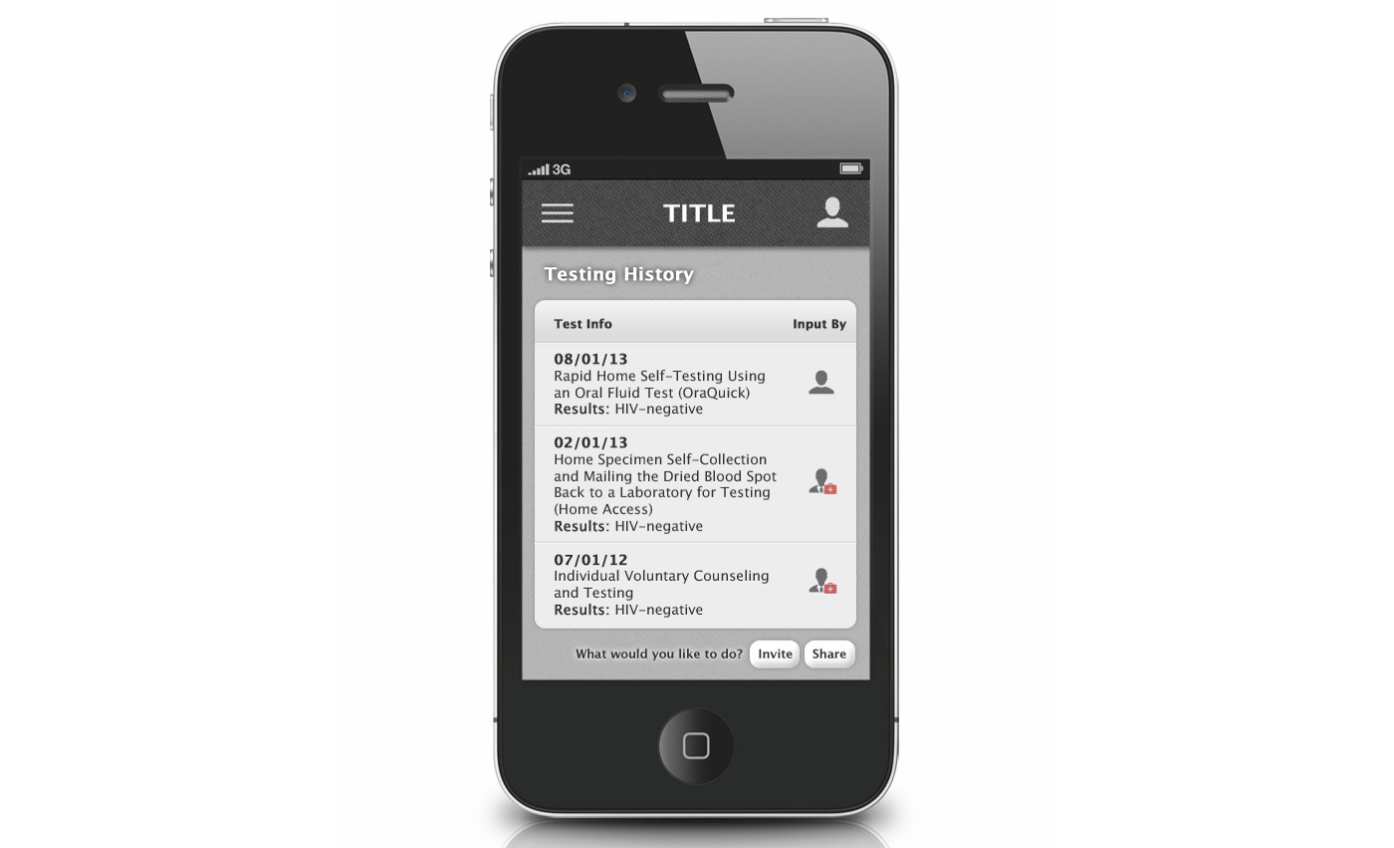
Screenshot for HIV testing documentation.

Each in-person FGD lasted approximately 1.5 hours, and the OFGD lasted approximately 2 hours. FGDs were conducted by 2 trained facilitators (one in Atlanta and one in Seattle) who were familiar with the goals of the mobile HIV prevention app. All FGDs addressed men’s general preferences for apps, HIV testing barriers and facilitators for MSM, and ways that an HIV prevention app could increase the frequency of HIV testing and prevention among MSM. During FGDs, participants were shown screenshots and provided feedback on eight potential app functions: (1) information about HIV testing options and creating a testing plan (Figure 1), (2) use of the phone’s calendar for reminders of upcoming HIV testing dates, (3) a map of HIV testing sites, (4) location-based reminders for HIV testing when near a testing center (Figure 2), (5) non–location-based reminders for HIV testing, (6) rating and reviewing HIV testing centers and other venues, including a review on how gay-friendly the venue is (Figure 3), (7) tracking of sexual behaviors over time with a summary describing results (Figure 4), and (8) documentation of HIV testing results (Figure 5). Participants also provided suggestions for how to improve each function and the app overall, identifying additional functions that should also be included.

### Data Analysis

In-person FGDs were audio-recorded and transcribed verbatim, and the OFGD was automatically downloaded to a readable text file. Analysis was conducted using MAXQDA, version 10. A team of 4 data analysts conducted a thematic analysis, examining both inductive and deductive themes within the transcripts. After multiple close readings, the team created a preliminary codebook of all salient themes. Provisional definitions were given to each code and 3 analysts applied each code to a single transcript. The coded transcripts were merged for comparison and code definitions were revised based on coding disagreements. Once the final codebook definitions were established, 3 data analysts consistently applied the codes to all of the transcripts. All transcripts were double-coded with 2 analysts each coding the same transcript. Transcripts were then merged and codes were reconciled; differences among coders were resolved by consensus.

After multiple purposeful and focused readings of coded text, thick descriptions were created for each theme. The descriptions identified common concepts, patterns, and unique ideas expressed in the FGDS. Themes were analyzed separately based on location and were compared and contrasted between groups.

## Results

### Overview

In total, 38 MSM (all identifying as gay or bisexual) participated in this study. Demographics varied based on location (Table 1). Participants in Seattle had an older mean age (39 years) than in Atlanta (29 years) or rural regions (30 years). Racial composition of participants varied; Atlanta was the only site with African American participants (35% of Atlanta participants, 6/17). Participants in Seattle were more likely to have ever taken an HIV test (Seattle: 91%, 10/11; Atlanta: 88%, 14/16; Rural: 70%, 7/10), but participants in Atlanta were more likely to have taken an HIV test within the past 3 months (Atlanta: 57%, 8/14; Seattle: 10%, 1/10; Rural: 14%, 1/7). Participants discussed 16 themes that were incorporated into the codebook (Table 2). There was some geographic variation in discussions of these themes, especially regarding preferences about the language and tone of the app. There was less variability for other preferences, such as the functionality and content of the app.

**Table 1:** Participant demographics.

		Atlanta (n=17)	Seattle (n=11)	Rural (n=10)	Total (n=38)
Age in years, mean (range)		29 (23-40)	39 (19-63)	30 (19-41)	32 (19-63)
**Race, % (n)**					
	White/Caucasian	58.8 (10)	80.0 (8)	80.0 (8)	68.4 (26)
	Black/African American	37.5 (6)	0.0 (0)	0.0 (0)	15.8 (6)
	Other	6.3 (1)	20.0 (2)	20.0 (2)	13.2 (5)
**Sexual orientation, % (n)**					
	Gay/Homosexual	87.5 (14)	90.9 (10)	90.0 (9)	86.8 (33)
	Bisexual	12.5 (2)	9.1 (1)	10.0 (1)	13.2 (5)
Has ever taken an HIV test, % (n)		87.5 (14)^a^	90.9 (10)	70.0 (7)	83.8 (31)
How many HIV tests have you had in the last 12 months? Mean (range)^b^		2.1 (1-4)	0.8 (0-3)	0.7 (0-2)	1.4 (0-4)
**How long ago was last HIV test** ^b^ **, % (n)**					
	<3 months ago	57.1 (8)	10.0 (1)	14.3 (1)	32.3 (10)
	3-6 months ago	35.7 (5)	30.0 (3)	28.6 (2)	32.3 (10)
	6-12 months ago	7.14 (1)	0.0 (0)	14.3 (1)	6.5 (2)
	>1 year ago	0.0 (0)	30.0 (3)	28.6 (2)	16.1 (5)
	>5 years ago	0.0 (0)	30.0 (3)	14.3 (1)	12.9 (4)
**Where received an HIV test, % (n)** ^b^					
	CBO	78.6 (11)	80.0 (8)	42.9 (3)	71.0 (22)
	Doctor’s office	71.4 (10)	50.0 (5)	42.9 (3)	58.0 (18)
	At home	21.4 (3)	20.0 (2)	14.3 (1)	19.4 (6)
	Other	14.3 (2)	20.0 (2)	14.3 (1)	16.1 (5)

^a^One participant did not answer, total n=16.

^b^Among participants who ever had an HIV test.

**Table 2:** Code definitions.

App preferences	General discussions about what participants like/do not like about the apps that they use
Beyond HIV testing	STI testing, PEP, PrEP, linkage to care (for HIV and STIs), mental health, other health issues; include discussions in support of and opposed to including additional information; include any discussion of HIV/STI prevention that goes beyond the original suggested purpose of the app; DO NOT include sex diaries, gay yelp
Credibility/ Authority	Discussions about trusting the app and the information that it is providing, credibility of the app and sources behind information (eg, CDC, doctors, universities), reliability of how the app reports results, whether or not an authoritative tone is more trustworthy, concerns regarding abuse of the app
Customization/ Personalization	Discussions of ways that the app can be customized or personalized to fit the needs of different app users; discussion of anything optional or described as “this should be optional”; Code when a participant states “this is not something that would work for me, but I can see this working for people in general” – statements that express how different users may want to interact with the app differently
Design/ Functionality	Any reference to the layout of the app, functions that should or should not be included in the app (and why), usefulness of functions, relevance of functions, comments about how cluttered it is, images, etc; ease of use, simplicity; statements describing if it is “simple”, “straight forward”, “confusing” etc; battery life, data usage
Education	All discussions of HIV/STI and health education/information, including discussions of how the app does or can educate, why education is important, etc; the type of education/information that participants want; how they want to receive education/information
Interactive engagement	Engagement with the app rather than simply receiving information (eg, discussions of putting information into the app via quizzes, diaries, etc), discussions of how the app already includes or can include more interactive engagement, the importance of interactive engagement, the impact of interactive engagement on motivation to use the app
Perceived impact	How participants described the impact that the app could have on HIV testing, HIV risk, and sexual health, including both the individual impact and the community impact; the ability for the app to help start conversations about HIV, the ability for the app to improve HIV prevention, HIV testing behaviors, safer sex behaviors, etc
Privacy/ Confidentiality	Any discussions/concerns about privacy, confidentiality, or security; concerns that insurance companies may get information about the app; legal concerns regarding HIV transmission
Relatable vs professional	Discussions of whether or not the tone/language of the app should be relatable or professional and why
Sharing data	Willingness or unwillingness to share data with the program developers; sharing data as a way to enhance the app, as a way to get information back about yourself and/or community, for research, as a way to give back to the community; any motivation for sharing data or not sharing data; DO NOT code when discussions of sharing on social networking sites
Social networking	Using the app for the purpose of networking with the others; “check-ins”; connections to Facebook, online dating sites, or other networking sites; using the app for communication with others in a social network, advertising on social networking and/or online hook-up sites, sharing information with individuals (friends, partners, etc)
Stigma	Discussions about HIV stigma and homophobia and how the app can impact stigma
Target population	Discussions about the target population and app audience demographics, including sexual orientation (and outness), age, race, rural vs urban, Spanish speakers, etc; comments about how “gay” to make the app, ie, “don’t make it too gay”
Testing barriers/ Facilitators	Discussions about current barriers and facilitators for regular HIV testing for MSM, including access to testing, knowing where testing sites are located, feeling safe/unsafe when going to get tested, anonymous testing vs required reporting, etc
Willingness/ Motivations	Expressions of willingness (and unwillingness or reluctance) to download and/or use the app, explanations of why willing (or unwilling) to download and/or use the app; statements about whether or not there is a perception that others would be willing to use the app, discussions of what would motivate (or not motivate) someone to download and use the app regularly

### Functionality

#### Education

In all FGDs at all study sites, participants recognized an educational component of the app as being vital to HIV prevention: “A real important aspect of prevention is education…it needs to have an educational element so that people who might be questioning whether or not they should go and get tested, they can” (FGD1, Atlanta). Participants identified the types of information that they would want to receive about HIV testing options, including education about accuracy of tests, where to get tested, home-testing (especially instructions on what to do if someone receives a positive test result after administering a home test), and information about window periods of HIV tests. This education about the types and availability of HIV tests was perceived as useful because it would help guide men in decision-making about which type of test is best for them:

For people who have maybe not got tested before, are not really confident about it, some sort of pro/con list for each [HIV test]… So someone just doesn’t have to look at this and make a decision on their own.

FGD2, Atlanta

I really like the take a [testing preference] quiz option. So if you have absolutely no idea, you could go through and it will prompt you for which one might be the best for you.

FGD2, Atlanta

Men also felt that education about HIV should go beyond HIV testing to include information about linkage to HIV care, sexually transmitted infection (STI) information, STI treatment, safer sex tips (especially among sero-discordant couples), pre-exposure prophylaxis (PrEP), non-occupational post-exposure prophylaxis (nPEP), and current HIV research. Men felt that this additional information would provide a more comprehensive education about HIV prevention and was perceived as more “relevant” to the general MSM population because it would benefit all MSM, regardless of HIV status.

Though education was considered important, participants also stated that an HIV prevention app would need to provide more than just information in order to engage app users:

Some sort of a reason to use the app is really important…having all the information on there is good but you could also Google HIV testing centers. I mean, it’s possible. The information wouldn’t all be in the same place. But having a reason to do it is good…with the testing options, if you could order from there, it would give you like a coupon for 10%, 15% off of a swab kit or something.

FGD1, Atlanta

#### Interactive Engagement

Participants stated that interactive engagement where app users needed to input information into the app (eg, through sex diaries) could help “keep people coming back using the app” (FGD2, Atlanta). Participants stated that the most frequently used apps involve some kind of “user input” that “gives somebody a reason to go into the app and do stuff on it… it’s not strictly information based” (FGD1, Atlanta). Based on this idea of increased engagement, many participants suggested interactive game-like functions as a more enjoyable way to receive information.

Although men were interested in functions that involved interactive engagement, they also recognized that inputting information into an app requires a lot of “commitment” and that the novelty of inputting the information could wear off after a period of time. To address this, participants stated that feedback from inputting information into the app would enhance engagement:

I downloaded the Kinsey survey app…it never gave you end results…it was interesting in a sense to me personally. I don’t use it as much anymore because the interest wore off because there was no actual use out of it. Something like this [HIV app] I would probably use because it would be interesting for me to see what the results turned into.

FGD2, Atlanta

Many participants described wanting community information to “compare it to everyone else that’s using the app” (FGD2, Atlanta):

I would love if these data were actually aggregated and researched…I would love to see a summary of my city and just in terms of averages or something…[For example], last month the average gay man in this zip code in Seattle had X number of different partners.

FGD4, Seattle

Participants also expressed wanting feedback about personal behaviors:

I also like this too to see statistics [based on your behaviors]…you were drunk or high 3 out of 10 times [that you had sex…that isn’t something that I would necessarily remember or think about. But then if you see something like this and it’s like you were drunk or high the past 9 out of 10 times, it’s like oh. I like to see where you’re at and just personal feedback where maybe some of the other apps might not actually let you see the results.

FGD2, Atlanta

Well I love seeing these kind of info-graphics and digestible things about me that I don’t know already so something that can tell me cool facts about me without me already knowing that in advance is kind of neat.

FGD4, Seattle

Participants felt that receiving this feedback on personal behaviors could help with HIV prevention by increasing self-awareness of one’s own HIV risk:

I think people will sometimes tell themselves this person seems clean, this person seems healthy, he says he doesn’t have HIV, we don’t have a condom as kind of ways to rationalize it. And then they could be recording and tracking all of that and an app would be able to tell them, “you’re engaging in high-risk sexual behavior.” And I think, as obvious as it might seem, like oh, you just had bareback sex last night with someone you met online. Of course you should realize, but people I think do that and we would have an app to be able to send that message. They’re sitting down and they say, this voice of science and authority in medicine says you are putting yourself at risk of exposure to HIV.

FGD2, Atlanta

It’s easy to say “I am an adult, I know what I’m doing,” but that isn’t always true. Some people NEED to know the truth about their choices.

OFGD, rural

App feedback was perceived as even more useful if men were then linked to services based on their behaviors; for example, participants suggested that if an app user reported a lot of unprotected sex, they could be provided with more information about HIV testing.

#### Social Networking

An additional recommendation for making the app more interactive was to incorporate social networking. Participants described using various types of social media (eg, Facebook, Twitter) as well as online hook-up sites and apps (eg, Grindr) and explained how social media could be used with HIV prevention. Some participants talked about sharing their status or sharing the fact that they got tested on sites like Facebook. Participants described how sharing this information through social media could encourage testing:

I think sharing it is a good way to get the word out and to encourage your friends to go or you to go. If you see that four friends got tested in the past week or two weeks or whatever, then you might be more inclined to go yourself.

FGD1, Atlanta

I think [posting that you got tested on Facebook] is a really good idea because every time I get tested, I post it on Facebook. I don’t post the results but I ask all my friends, do you know if you’re HIV positive or not? I just got tested and I usually give the address of where I went.

FGD2, Atlanta

Posting information about testing on social media was also perceived as a way to reduce stigma around HIV and MSM:

It might be nice to…have the option of putting out a message on Twitter or Facebook so you can say, “I just got tested, I know my status”…a lot of the voting, blood drives, they give you a little sticker that says I just did something and you feel good about it because you were responsible, did something you were supposed to do. And I think there’s usually not a lot of that around HIV testing because of the stigma around HIV or around men who sex with men when in reality, there’s lots of people out there getting HIV tests all the time. And it could be a social message to put out there.

FGD2, Atlanta

#### Language and Tone

Participants identified a need to present information in the app using simple language that is straightforward and concise: “I’m just thinking of things for lower intelligence levels, like writers for the newspaper are supposed to write on a fourth grade level” (FGD1, Atlanta). There was disagreement about the preferred tone of an app, with participants explaining the importance of two preferences for language: friendly and sexy versus professional and authoritative. In Seattle, participants proposed using sexier and “playful” language and content:

What is it that would make it fun to use or what would be a way to deliver the information in a way that people would actually want to digest it?…I’m thinking of a very extreme idea, but…what is it that gay men like? Well, they like sex. They like porn…what if instead of a clinician delivering the information, what if you actually have a video of two hot guys having sex and one guy is talking to the camera and he’s showing someone how to put on a condom properly and it’s not some clinical bullshit, it’s a hot guy with a hard on about to have sex, putting on a condom the right way.

FGD3, Seattle

Friendly, fun, and humorous language that is more subtle when addressing HIV and risk behaviors was described as less stigmatizing and less judgmental:

These [risky behaviors] are public health concerns and they need to be subversive and they need to be kind of a joke…because I don’t want to be preached at. As a gay man, I am subjected to enough external guilt about what I do and how often I do it and how I do it and with whom that I really don’t want extra guilt about I haven’t been tested in however long or I’m engaging in risky behaviors because the purpose is not to criticize, the purpose is to change the behavior and to get people the health care that they need. So I think you really have to put a premium emphasis on being sneaky about it and subversive.

FGD3, Seattle

On the other hand, participants in all locations identified wanting respected and trustworthy information and language: “We don’t want something ‘cute’ we want something authoritative…something that promotes security and trust” (OFGD, rural). Authoritative language was perceived as increasing the credibility of the app and the information that the app provides.

#### Target Population

Participants expressed differing opinions regarding the app’s target population with variation occurring within cities and within groups. Some participants recommended functionality specifically targeting gay men, while others expressed concerns about making the app “too gay”. Men felt that if the app targets gay men, then it would exclude some men and “would make it definitely not attractive to the bisexual/straight community” (OFGD, rural). Multiple participants across FGDs stated that if the app has a more general target population, then it may reach a wider population of men who may not be getting tested for HIV:

I just want to bring up a point…for lack of a better word, not to make this too gay, I think a lot of the problem of people not getting tested is they think HIV is a gay disease… A lot of people who don’t have that information are probably more in the closet or they live somewhere where they can’t be who they are or they’re not gay and they’re something else on the spectrum and they don’t want to necessarily read articles from [an Atlanta LGBT news source] or something similar on this sort of app…it can focus on that LGBT community but maybe not be so overt about it, just to encourage as many people to use it as possible.

FGD1, Atlanta

Some participants identified the app as “universal” and therefore suggested that it should not specifically target the gay community. Men suggested that the app could be advertised for gay men and used by gay men, but that the app itself does not need to have content or language that exclusively targets the gay community.

Some rural men especially felt that the app should not target just gay men and expressed concerns that this would be “discriminatory” and “make gays feel more in the spotlight” (OFGD, rural). However, rural men also felt that targeting MSM was important because it “targets the high risk area” and “it is targeted to those who will most likely be looking for the information” (OFGD, rural). Participants in other FGDs also identified the importance of including content that is specifically aimed at gay, bisexual, and other MSM, such as providing resources on gay-friendly testing locations, providing health information on gay sex, incorporating gay blogs or local gay news sources, aggregating collected data to provide fun HIV- and sex-related statistics on the gay community etc.

### Willingness to Use the App

#### Overview

Participants varied in their willingness to use the app and share data; this variation occurred both between and within groups and study sites. Some men said that they would not be willing to share very personal and private data with the app, especially data related to sexual experiences and HIV status:

I think the information that [the app] is asking is way too much, private. I would never submit those kind of information to an app. I don’t trust the privacy of that. If you ever put your email address in there, you never really know.

FGD1, Atlanta

Uncertainty about inputting information and sharing data were specifically related to privacy and concerns about who would be able to access the information. Some men were concerned about the potential for negative consequences if private health information or data about sexual experiences were accessed by others:

To me it looks like an information-gathering thing…almost like a big brother. This government has access to everything on your phone in one way or another…But if your insurance starts to deny you based on you being too promiscuous because they’ve got information on the average gay men has sex however many times a month. And that’s what it’s going to get to when the prices of insurance start going up and things like that. It gets more and more difficult. They already do it with cars. You know, you can get an insurance rate based on that little thing you plug under your dash.

FGD1, Atlanta

However, many participants also expressed a willingness to use the app and share data. Participants stated that men may be willing to share data if the app promotes altruistic motivations for engagement. One such form of altruistic motivation is sharing data for the purpose of research or to help health organizations: “I think the value of this information…if you opted to share that information anonymously that would be very helpful for health organizations to know what’s going on” (FGD3, Seattle). Men also suggested using altruistic motivations by providing financial incentives to HIV organizations when men input and share data with the app:

I think altruism is a good thing to build with apps. There’s one on My Quiz where…for every question you get right they donate a pound of rice to the third world or something like that. So, facts about HIV, whenever you do it there’s a donation made by one of the sponsors to the AIDS Foundation or some research association…I think the altruism could be a way to incorporate more engagement.

FGD4, Seattle

#### Privacy and Discretion

Though participants expressed many concerns about privacy, some participants explained that if they trusted the app and the app’s creator, then they may be more willing to share private information. Men also explained the importance of discretion with an HIV prevention app aimed at MSM: “There’s a degree of discretion that someone might want with the content…If they’re looking at these things…they’re not going to be doing it just at home” (FGD3, Seattle).

One suggestion was to be careful about icons and language, so that if others were to gain access to an app user’s phone, they would not identify what the app is:

I could imagine if someone gets an HIV positive result, they’re not going to want that to be something that oh, my little sister picks up my phone and sees this. So I would just be very thoughtful about how you designed those features…I think that would be critical to make sure that that’s done in a way that minimizes the risk of any type of exposure that people don’t want.

FGD2, Atlanta

I’ve had friends ousted on various social media and apps so and even just having the icon of Grindr on someone’s phone, it’s a very distinct tell…I can only imagine if I wasn’t out that would be something that I would be very concerned. I don’t know if I would keep an app like that on my phone at all, just because I wouldn’t want to be found.

FGD4, Seattle

Discretion was considered to be especially important when sending push notifications to the phone:

I think the wording of [push notifications] would be pretty important not to have anything about HIV testing or something pop up on your screen. Your phone could be wherever.

FGD1, Atlanta

I am often times in meetings and it’s often me who’s projecting up on a giant computer. The last thing I want is the schedule plus alert saying that it’s time for me to get an AIDS test.

FGD3, Seattle

Participants also recognized the importance of password-protected data and suggested using a separate password for the app*.*

#### Perceived Impact of the App

Participants discussed a perceived impact that the app could have for individuals who use the app, for sexual partnerships, and for the MSM community as a whole. For individuals, men recognized that the app could be a useful personal tool that “provides a lot of accountability” (FGD2, Atlanta) for one’s sexual health and sexual decision-making, increases personal awareness and “self-analysis” of one’s behaviors, assists with ownership over health behaviors, and could help men make “active attempts to stay healthy” (FGD3, Seattle). As a way to promote self-care for individuals, participants recognized that the app could have a useful impact by connecting individuals to resources, tracking sexual behaviors, and tracking HIV test results; these functions could help to increase self-awareness and could be “psychologically useful” (FGD3, Seattle). In this regard, participants saw the app as doing more than just encouraging HIV testing; they perceived it as a “useful life tool” (OFGD, rural). As a link to resources, participants identified the app as useful to all MSM, but also as something that could be especially useful to MSM who live in regions that do not have a lot of gay-friendly HIV services: “I think in some places, the places that are more rural, this would actually be even more useful because there’s less general knowledge of services” (FGD2, Atlanta).

The app was also seen as a useful way to discuss HIV with partners and friends and to help identify others who are also “taking the proactive steps” (FGD1, Atlanta). Participants stated that if others had this app on their phone, then they would not necessarily assume that they were HIV-negative, but they would identify that person as someone who is “more responsible” in terms of their sexual health. Men also stated that the app could be used as a tool for conversations with partners about HIV and that men would like to share dates of HIV tests and results with their partners through the app.

For the community, participants recognized that an HIV prevention app could help promote a culture where self-care around sexual health is a priority and is normalized: “If the goal of the [calendar and reminder] function is to promote a new culture where the testing is part of our self-care, [the function] is important because it’ll encourage the culture to start to form over time” (FGD3, Seattle). Participants also discussed the importance of the app in promoting a nonjudgmental and sex-positive space for men to discuss issues of sexual health within communities:

The gay community that really needs something like this is very social about this conversation and having like a little Facebook where everybody gets to log on and call each other sluts would actually be fun and for the people who really need this functionality, they’re talking about it amongst each other anyway, I think.

FGD3, Seattle

The app was not perceived as something that would exist on its own, but rather a tool that could be used in conjunction with the HIV prevention efforts that are already occurring in the MSM community:

We used to go out to the bars with literally safety pins and hand them out and give them to people and it was something that as a community we did as an outreach to educate people…but I don’t see a lot of that, and when I see a profile of somebody that’s 22 years old that’s HIV positive online, my thinking is what are we doing as a community that we failed this person?...it’s a hard disease to get and it’s easy to avoid and if you just have the right information. And I think that we’re not getting that out there…so this app really what it should be doing is augmenting what we as a community are already doing, which means we as a community maybe need to also think about how we can deal with this because it boggles my mind. 22 years old and HIV…we celebrate birthdays as a community, we sober up as a community, we celebrate momentous times in our life as a community, so why are we not coming together for this?

FGD3, Seattle

## Discussion

### Principal Findings

In summary, participants described a comprehensive app that should incorporate innovative ideas to educate and engage men to increase motivation to use the app. Participants also suggested using existing social media platforms to engage MSM in HIV prevention. In order for an app to be useful, it needs to feel safe and trustworthy, which is essential when considering the app’s language and privacy. Regardless of precautions that may be taken, some men may not feel comfortable inputting personal information in an app; however, if the app proves to be credible and has safeguards to ensure discretion and privacy, then MSM may be more willing to use it. Men also expressed a willingness to share data anonymously if it would contribute to research about their community or help AIDS service organizations. These findings suggest that if an HIV prevention app can be developed so that MSM will be motivated to use it, then it may be able to simultaneously address individual, interpersonal, and community-based needs for HIV prevention.

Our findings are similar to results from other studies examining MSM’s preferences for app use. Others have identified similar desires for sex education and links to resources, such as STI and HIV testing, gay-friendly providers, and resources for MSM who are living with HIV [26-28]. Participants’ discussions of the use of social networking for HIV prevention augment previous reports about effective interventions based in social media sites, such as Facebook [29,30]. These studies use Facebook as a means to provide an intervention, but participants in this study suggested using an app that links with existing social media to encourage increased use. Expressed preferences also align with guidelines for mHealth practices [31], which suggest that mHealth interventions use scalable platforms, offer sustainable possibilities, address a willingness for app use, encourage continued engagement, provide connections to social networks, and measure social network and/or geographic data [31].

Many of the participants’ suggestions are based on what they want in an app, but this often also aligned with what men might need for HIV prevention; for example, participants expressed a desire for increased accountability to improve HIV risk-reducing behaviors and encouragement for increased HIV testing. Many of the participants’ suggestions also addressed how to make the app more fun (eg, games, sexy content). Although these suggestions might not be as directly related to what men need for HIV prevention, they are still useful. Making an HIV prevention app enjoyable and usable for MSM is a challenge; however, the usability of the app is vital because no matter how great the intervention, if MSM do not use the app, then it will not be useful.

Identifying the appropriate language to make an app enjoyable yet usable may be challenging, as sometimes participants’ suggestions were contradictory. Participants expressed wanting the language and tone of the app to simultaneously be professional, credible, and trustworthy, while also including language that is more sexy, fun, and nonjudgmental. This variety in app preferences, especially regarding language, identifies a need for customizable app options. One possibility for addressing these contradictory app preferences is to build the app using two different options for language and allow app users to customize their app by choosing the voice or tone that they want their app to have. However, this option may not be feasible as it would require twice as much work for building the app. A more cost-effective option may be to incorporate more formal or clinical language for some features (eg, for a description of HIV tests), but more informal or conversational language for other features (eg, for behavioral assessments). Based on these findings, we learned that it is important to find the right balance of language in the app so that it is sexy and fun, but not so much that it discredits the feeling of authority of the app. According to participants, it is also important that the language be nontechnical and easy for anybody to understand. Formal language should still be simple and nonjudgmental.

### Limitations

There are some limitations in this study. These qualitative findings may not be transferrable to a larger population of MSM. Only 16% of participants in this study identified as black/African American, all of whom were in the Atlanta FGD. The greater recruitment of African American MSM in Atlanta is reflective of Atlanta’s population [32], but more direct targeting for young African American MSM may have been useful to ensure the inclusion of the population most at risk for HIV [1,33]. Furthermore, MSM who identify as gay or bisexual and include that they are seeking other men on Facebook may not represent MSM in general. However, recruitment occurred in two different cities where populations, culture, and HIV efforts vary. This study also included rural MSM nationwide; rural MSM face unique challenges related to HIV, such as a lack of resources or increased stigma [34-39]. We found some geographical variation in responses but also agreement among participants in different locations. Additional FGDs, especially with rural men, may have provided greater variation. FGDs with rural men were limited by the online environment. Men participating in these groups needed to have access to a computer with Internet. Furthermore, the facilitator was unable to use nonverbal cues to assist with probing questions. Despite these limitations, the OFGD was useful for capturing a population that would have otherwise not been able to participate in this study and the OFGD supplemented the in-person FGDs by highlighting the similarities and differences in opinions that MSM have in other regions throughout the United States.

### Conclusions

Bringing HIV prevention services to scale for MSM is a critical prevention priority [5,40]. At a time when mobile phone use has become the norm in the United States [7] and mHealth is advancing and becoming a more popular medium for HIV prevention interventions [11,22], it is important to understand preferences for mobile apps to deliver public health interventions. In order for an HIV prevention app that targets MSM to be useful, it needs to address the challenges and barriers that MSM face with HIV testing and HIV prevention, while also appealing to the community as a fun, trustworthy, and easy-to-use app. Participants suggested that if successful, this type of intervention could have a great impact on HIV prevention. However, in order to be successful, additional efforts must be made to address MSM’s wants and needs regarding HIV prevention and interventions based in mobile technology.

## Abbreviations

CDC: Centers for Disease Control and Prevention
FGD: focus group discussion
HIV: human immunodeficiency virus
MSM: men who have sex with men
OFGD: online focus group discussion
nPEP: non-occupational post-exposure prophylaxis
PrEP: pre-exposure prophylaxis
SMS: short message service
STI: sexually transmitted infection
